# NHE9 induces chemoradiotherapy resistance in esophageal squamous cell carcinoma by upregulating the Src/Akt/β-catenin pathway and Bcl-2 expression

**DOI:** 10.18632/oncotarget.3618

**Published:** 2015-04-10

**Authors:** Junying Chen, Hong Yang, Jing Wen, Kongjia Luo, Qianwen Liu, Yijie Huang, Yuzhen Zheng, Zihui Tan, Qinyuan Qingyuan Huang, Jianhua Fu

**Affiliations:** ^1^ Sun Yat-sen University Cancer Center, State Key Laboratory of Oncology in South China, Collaborative Innovation Center for Cancer Medicine, Guangzhou, China; ^2^ Guangdong Esophageal Cancer Institute, Guangzhou, China; ^3^ Guangdong General Hospital, Guangdong Academy of Medical Sciences, Guangzhou, China

**Keywords:** NHE9, esophageal cancer, chemoradiotherapy, Src, Bcl-2

## Abstract

Recently, we found that NHE9 mRNA was upregulated in chemoradiotherapy (CRT)-resistant esophageal squamous cell carcinoma (ESCC); however, the underlying mechanisms were unclear. Here, we aimed to clarify the functional contribution of NHE9 to CRT resistance, understand the molecular basis of NHE9-dependent resistance in ESCC, and identify potential therapeutic targets. Our results showed that NHE9 prevented CRT-induced apoptosis. Importantly, we found that RACK1 is a novel binding partner of NHE9 and that NHE9-dependent induction of CRT resistance requires the activation of RACK1-associated Src/Akt/β-catenin signaling. Moreover, upregulated Bcl-2 protein was also observed in cells exhibiting NHE9-induced CRT resistance. A higher NHE9 level was associated with a poor response to CRT and less decrease in T and N stage in ESCC patients. Furthermore, combining either Dasatinib or ABT-737 with CRT significantly reduced tumor volume, and the response to CRT was restored when these inhibitors were used together with CRT in a xenograft nude mouse model with NHE9 overexpression. Taken together, our findings demonstrate that NHE9 can be an effective predictor of CRT response and may be useful in the development of targeted therapies for CRT-resistant ESCC.

## INTRODUCTION

Currently, it is clear that neoadjuvant chemoradiotherapy (CRT) can improve the outcome of esophageal squamous cell carcinoma (ESCC) [1, 2]. However, only responders benefit from CRT, whereas nonresponders may suffer a worse prognosis [3, 4]. Therefore, it is very important to explore the key markers and relevant subcellular processes affecting the CRT response. We previously conducted a microarray study using mRNA extracted from tumor tissue obtained from ESCC patents via endoscopic biopsy before CRT to examine differences in gene expression between responders and nonresponders [5]. Among the differentially expressed genes, the NHE9 gene, which is implicated in endocytic recycling, was the only gene in this group that was significantly upregulated: its level in the responder group was two-fold higher than that in the nonresponder group. Therefore, it is possible that NHE9 is involved in regulating the response of ESCC to CRT; however, this requires further investigation.

NHE9, which belongs to the Na^+^/H^+^ exchanger superfamily, localizes on late recycling endosomes and maintains cation and volume homeostasis through electroneutral exchange of protons for Na (+) across membranes [6]. Members of the NHE family contain a long intracellular C-terminus that may interact with various proteins, such as calcineurin-homologous proteins (CHP1 and CHP2) [7, 8], receptor for activated kinase C (RACK1) [9, 10], calmodulin (CaM) [10, 11], and PIP2 [12], all of which play key roles in regulating cell function via various signaling pathways. Nevertheless, the interactions between NHE9 and these proteins have not been confirmed. NHE9 was reported to be associated with autism susceptibility and attention-deficit/hyperactivity disorder [13, 14]. Currently, the exact role of NHE9 in the CRT response of ESCC has not yet been determined, and despite the expression difference, the mechanism underlying NHE9 involvement in CRT resistance is unclear. However, considering the many potential interacting proteins, NHE9 could alter signaling pathways and ultimately cause ESCC cells to become resistant to CRT.

A growing body of evidence has shown that aberrant activation/sequestration of signaling pathways such as PI3K/Akt [15–17], MAPK [18, 19], and TP53 [20, 21] is correlated with the inhibition of apoptosis that is often observed in cancers with acquired resistance to treatment [16, 22, 23], [24, 25]. Several potential binding partners of the NHE family, including RACK1, CHP1, CHP2, CaM and PIP2, participate in regulating apoptosis-related cellular processes and in establishing crosstalk between intrinsic and extrinsic apoptotic pathways [24–28]. For example, RACK1 acts as an IGF-1R-interacting protein to activate Src/Akt and promote cell survival [29]. Additionally, CHP1 and CHP2 can respectively activate and inhibit the phosphatase activity of calcineurin [28], which induces apoptosis by enhancing BAD heterodimerization with Bcl-xl [30]. Therefore, NHE9 may regulate the response to CRT by interacting with key factors in various cellular processes. In this study, we explored the possible functions and corresponding mechanisms of the NHE9-regulated response to CRT in ESCC, and sought potential therapies that may offset NHE9-induced resistance.

## RESULTS

### NHE9 increases CRT resistance in ESCC cell lines

**Figure 1:**
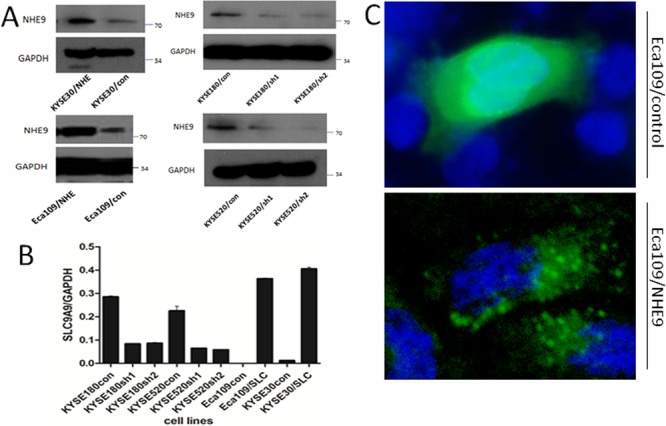
NHE9 overexpression or knockdown cell lines NHE9 was stably overexpressed in Eca109 and KYSE30 cells; NHE9 was stably knocked down in KYSE520 and KYSE180 cells; **A.** The efficiency of overexpression and knockdown was re-examined by real-time qPCR **B.** Subcellular localization of GFP-fused NHE9 compared with control GFP protein in Eca109 cells **C.**

We examined the expression of NHE9 in ten ESCC cell lines (Figure S1), and we selected Eca109 and KYSE30, which have relatively low levels of intrinsic NHE9, to establish stable overexpression lines (Eca109/NHE9 and KYSE30/NHE9). Inversely, KYSE520 and KYSE180, with relatively high levels of intrinsic NHE9, were used to generate stable knockdown cell lines (KYSE520/sh1, KYSE520/sh2; KYSE180/sh1, KYSE180/sh2). Subsequently, the efficiency of overexpression and knockdown was confirmed by real-time PCR and western blotting (Figure 1A–1B). Additionally, the overexpression of NHE9 was also confirmed by an immunofluorescence assay (Figure 1C). GFP-fused NHE9 was mainly localized on the membranes of endosomes and in the cytoplasm, whereas the control GFP protein was dispersed throughout the cytoplasm and nucleus.

**Figure 2:**
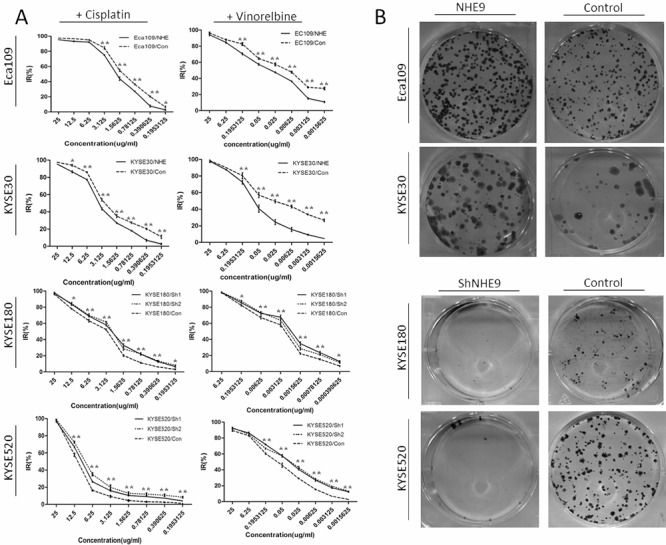
NHE9 downregulates the CRT sensitivity of ESCC cell lines Cisplatin and vinorelbine inhibition curves revealed that the IC50 of these drugs was significantly elevated in Eca109/NHE and KYSE30/NHE cells and significantly decreased in KYSE520/Sh and KYSE180/Sh cells **A.** *P < 0.05, **P < 0.01). Eca109/NHE9, Eca109/Con, KYSE30/NHE9, KYSE30/Con, KYSE180/Sh, KYSE180/Con, KYSE520/Sh, and KYSE520/Con cells were incubated for two weeks after X-ray treatment (6 J/m^2^). Photographs of crystal violet-stained colonies revealed that the radiation resistance in NHE9-overexpressing cells was significantly increased, while the resistance in NHE9-knockdown cells was significantly decreased **B.** All experiments were performed at least three times with the same results.

Both the overexpression and knockdown cell lines were treated with cisplatin or vinorelbine to study the possible function of NHE9 in regulating the response to chemotherapy. A series of 17 drug concentrations, ranging from 25 μg/ml to 3.9 × 10^−4^ μg/ml, were established using the double ratio dilution method and utilized to calculate the IC50 dose of cisplatin or vinorelbine in each cell line. The results showed that the IC50 doses of both drugs were significantly higher in the NHE9-overexpressing cells than in the corresponding controls; inversely, NHE9 knockdown obviously reduced the IC50 dose of both cisplatin and vinorelbine (Figure 2A). The IC50 doses of cisplatin and vinorelbine for each cell line and the relevant *P* values are listed in Table 1.

**Table 1:** NHE9 upregulates the IC50 dose of cisplatin and vinorelbine in ESCC cell lines

Cell lines	IC50 dose (μg/ml)		*P* value#	
	CP^1^	NV^2^	CP	NV
Eca109/NHE	1.7	3.0 × 10^−2^	<0.01	0.01
Eca109/Con	1.1	1.7 × 10^−2^		
KYSE30/NHE	3.1	7.1 × 10^−2^	<0.01	0.02
KYSE30/Con	2.5	1.6 × 10^−2^		
KYSE520/Sh1	7.6	3.8 × 10^−2^	<0.01	<0.01
KYSE520/Sh2	7.5	3.5 × 10^−2^		
KYSE520/Con	8.1	11.8 × 10^−2^		
KYSE180/Sh1	2.8	2.3 × 10^−3^	<0.01	0.01
KYSE180/Sh2	2.8	2.5 × 10^−3^		
KYSE180/Con	3.6	3.1 × 10^−3^		

1 CP = Cisplatin;

2 NV= Vinorelbine;

# Student's *t*-test results

An X-ray survival assay was performed on both overexpression and knockdown cell lines to examine the possible regulatory effect of NHE9 on the response to radiotherapy. Colonies were counted two weeks after x-ray radiation (Figure 2B). Fewer colonies were observed in NHE9 knockdown cell lines compared with the controls; in contrast, NHE9-overexpressing cell lines formed more colonies than the controls. KYSE180 was more sensitive to radiation because this cell line formed fewer colonies that were smaller in size compared with other ESCC cell lines treated with the same X-ray dose.

### NHE9 inhibits CRT-induced apoptosis

Although NHE9 was found to be capable of regulating the response of ESCC to CRT, the underlying mechanism was unclear. To abolish CRT resistance in patients with higher NHE9 expression, it is important to first identify the functional role of NHE9 in the response to CRT. We initially hypothesized that NHE9 might enhance CRT resistance by promoting cell proliferation and/or metastasis. This hypothesis was based on immunohistochemistry (IHC) results indicating that NHE9-positive cells were mostly observed in tumors, in the basal epithelium, and around microvessels. Subsequently, the growth curves, xenograft tumor sizes, and migration ability were compared between NHE9-overexpressing cells and control cells. Unfortunately, no apparent differences were observed in the cell growth curves (Figure S2A), and the tumor volume was similar between Eca109/NHE and Eca109/Con grafts (Figure S2B). Additionally, NHE9 did not exhibit effects on cell migration in a wound-healing assay (Figure S2C).

**Figure 3:**
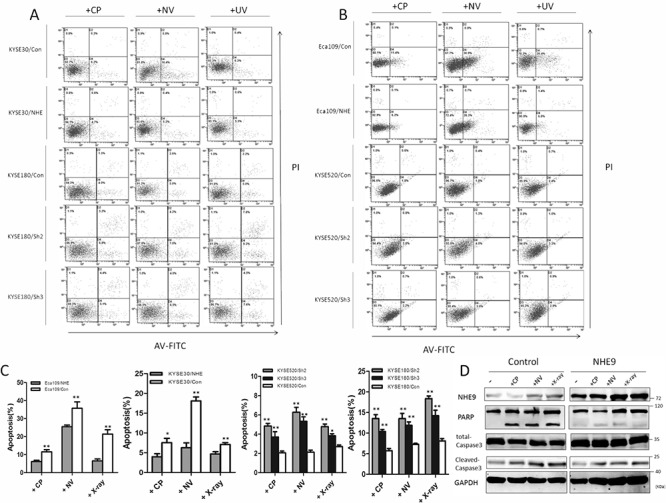
NHE9 downregulates chemoradiotherapy-induced apoptosis in ESCC cells A flow cytometry assay demonstrated that apoptosis was inhibited in NHE9-overexpressing cells, whereas increased apoptosis was observed in NHE9 knockdown cells. The data presented are the average apoptosis percentage **A–C.** *P < 0.05, **P < 0.01). Overexpression of NHE9 inhibits caspase-3 and PARP cleavage induced by anti-cancer drugs and X-ray exposure **D.** All experiments were performed at least three times with the same results.

To study the mechanism underlying NHE9-induced CRT resistance, we further tested whether the overexpression of NHE9 was correlated with decreased susceptibility to apoptosis induced by chemotherapy and/or radiotherapy. Flow cytometry was used to quantify cell apoptosis after cisplatin, vinorelbine or X-ray treatment. Obviously, Eca109/NHE9 and KYSE30/NHE9 cells exhibited significantly lower rates of apoptosis after anti-cancer drug or X-ray treatments compared with the controls, whereas NHE9 knockdown cell lines displayed a higher apoptosis rate (Figure 3A–3C).

Furthermore, the effect of NHE9 on apoptosis-related pathways was tested by western blotting, and the results revealed that NHE9 could inhibit CRT-induced apoptosis. The levels of cleaved-PARP and cleaved-caspase-3 in Eca109/NHE9 and KYSE30/NHE9 cells were much lower than those in the controls after chemotherapy and X-ray treatments (Figure 3D). These findings suggest that NHE9 induces CRT resistance by inhibiting cell apoptosis.

### Identification of RACK1 as a novel binding partner of NHE9 in ESCC

**Figure 4:**
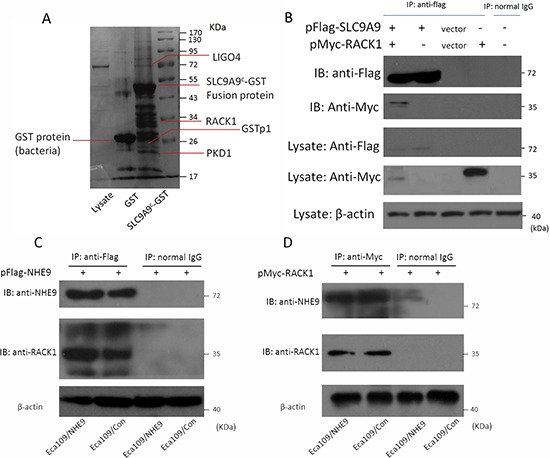
Interaction between NHE9 and RACK1 A pull-down assay suggested that NHE9 has four potential binding partners **A.** A co-immunoprecipitation assay confirmed that RACK1 is a binding partner of NHE9 in ESCC cells **B.** The interaction of NHE9 and RACK1 was further confirmed by immunoprecipitation using anti-Flag **C.** or anti-Myc **D.** beads. All experiments were performed at least three times with the same results.

Although NHE9 can induce CRT resistance in ESCC by inhibiting cell apoptosis, the underlying mechanisms by which NHE9, an ion channel, affects apoptosis are unknown. NHE9 is known to contain binding sites in its intracellular C-terminus for many molecules; therefore, we hypothesized that NHE9 might play a role in apoptosis through its binding partners. A pull-down assay was performed using the C-terminus of NHE9 (generated via prokaryotic expression) fused to GST-Sepharose and Eca109 cell lysates. The protein complex was further analyzed by SELDI-TOF-MS, and 4 potential interacting proteins, including *Homo sapiens* glutathione S-transferase pi 1 (GSTP1), leucine-rich repeat and immunoglobulin-like domain-containing receptor-interacting protein 4 (LIGO4), polycystin-1 (PKD1), and receptor for activated C kinase 1 (RACK1) (Figure 4A), were identified.

All four of the potential binding proteins were further identified by co-immunoprecipitation followed by western blotting; however, only RACK1 could be confirmed as a binding partner of the NHE9 C-terminus. Immunoprecipitation of NHE9 and RACK1 was applied to further confirm this result (Figure 4B–4D).

### NHE9 inhibits apoptosis by activating Src/Akt/β-catenin signaling

**Figure 5:**
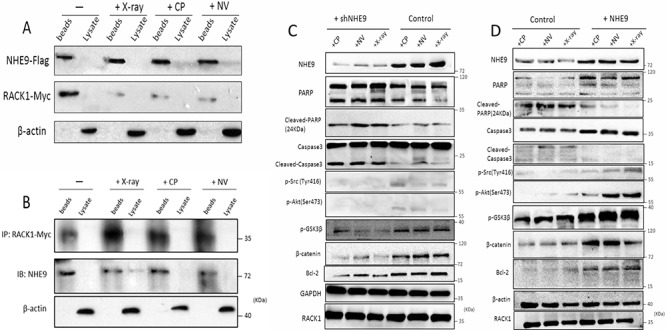
NHE9 inhibited apoptosis by activating Src/Akt/β-catenin and upregulating Bcl-2 The binding affinity of NHE9 for RACK1 changed after chemotherapy or radiotherapy **A–B.** Phosphorylated Akt (Ser473), phosphorylated Src (Tyr416), GSK3β, Bcl-2, and β-catenin were upregulated in NHE9-overexpressing cells when compared with the corresponding control cells **C.** Phosphorylated Akt (Ser473), phosphorylated Src (Tyr416), GSK3β, Bcl-2, and β-catenin were downregulated in NHE9 knockdown cells when compared with the controls **D.**

Interestingly, we found that the interaction between NHE9 and RACK1 was altered after the cells were treated with cisplatin, vinorelbine or X-rays. The immunoprecipitation results showed that the binding of NHE9 to RACK1 was relatively weaker after chemotherapy or radiotherapy, and a similar result was observed after X-ray treatment (Figure 5A and 5B). Therefore, CRT may negatively affect the binding between NHE9 and RACK1. Additionally, NHE9 may affect cell signaling activation by interacting with RACK1.

Because RACK1 downregulats cell apoptosis by regulating Src/Akt activity, we first examined the activation states of Src and Akt after anti-cancer treatment or X-ray treatment in NHE9-overexpressing and NHE9 knockdown cells. The western blot results showed that higher levels of phosphorylated Src (Tyr 416) and higher levels of phosphorylated Akt (Ser473) were detected in NHE9-overexpressing cells, whereas lower levels of phosphorylated Src and Akt were observed in NHE9 knockdown cells (Figure 5C).

We further evaluated the states of the downstream targets of Src/Akt. Activated Akt can phosphorylate and inactivate GSK3β, leading to the activation of β-catenin [31, 32]. To determine whether Akt/GSK3β/β-catenin was involved in NHE9-induced CRT resistance, the expression levels and phosphorylation states of GSK3β and β-catenin were evaluated. The results revealed a significant increase in Akt phosphorylation with concomitant upregulation of GSK3β and β-catenin in a cell model with NHE9 overexpression-induced CRT resistance (Figure 5C). Phosphorylated Akt was also suggested to be capable of upregulating Bcl-2 [33, 34], a potent anti-apoptotic molecule. Thus, we subsequently examined the expression of Bcl-2. Increased expression of Bcl-2 was detected in NHE9-overexpressing cells, which is reflective of resistance to treatment; inversely, relatively lower expression of Bcl-2 was found in NHE9 knockdown cells (Figure 5D). Collectively, our data indicate that NHE9 might induce CRT resistance by upregulating Src/Akt/β-catenin pathway and Bcl-2 protein expression.

### Inhibition of NHE9-induced resistance by Src and Bcl-2 inhibitors

Given that the aberrant activation of Src/Akt/β-catenin and the upregulation of Bcl-2 were considered to be involved in NHE9-induced CRT resistance in ESCC, we attempted to weaken the resistance by applying the relevant targeted inhibitors to decrease the activation of Src and Bcl-2. Dasatinib and ABT-737 are targeted inhibitors that are widely used for inhibiting Src and Bcl-2 activation, respectively, in basic research and clinical trials [35–38]. Therefore, a combination therapy including Dasatinib, ABT-737 and chemotherapy (consisting of cisplatin and vinorelbine) was applied in a nude mouse xenograft model to test its therapeutic effect on tumor grafts with NHE9-induced CRT resistance. The therapeutic effect of a combined regimen including Dasatinib, ABT-737 and X-ray radiation was also tested.

**Figure 6:**
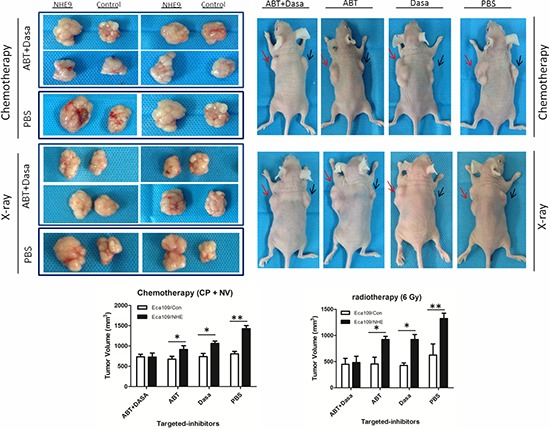
Inhibition of NHE9-induced resistance by Src and Bcl-2 inhibitors Representative image of the tumors formed in a nude mouse after the injection of Eca109/NHE (right flank, red arrow) and Eca109/Con (left flank, black arrow) and following intraperitoneal delivery of chemotherapy (or X-ray exposure) plus PBS, chemotherapy (or X-ray) plus ABT-737, chemotherapy (or X-ray) plus Dasatinib, and chemotherapy (or X-ray) plus ABT-737 plus Dasatinib (*P < 0.05, **P < 0.01).

No incidental death was observed during the experiment. The results demonstrated that the combined use of targeted inhibitors significantly reduced tumor volume (Figure 6). The volume of Eca109/NHE-formed grafts was reduced after treatment with Dasatinib plus chemotherapy or ABT-737 plus chemotherapy compared to chemotherapy alone; moreover, the response to CRT was restored when applying two inhibitors-plus-chemotherapy treatment in Eca109/NHE-formed grafts. The combined targeted-therapy had similar therapeutic effects in the group exposed to X-ray radiation.

**Figure 7:**
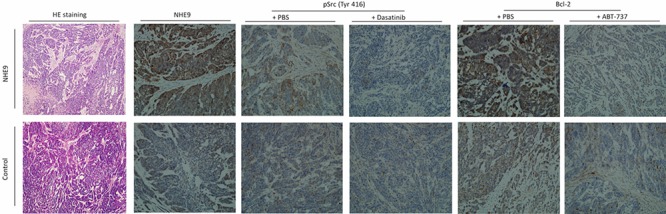
Representative pathological images of the xenograft HE staining of injected tumors confirmed the ESCC phenotype in both Eca109/NHE and Eca109/Con grafts. IHC staining for NHE9 showed that the expression of NHE9 was significantly higher in Eca109/NHE grafts. Phosphorylated Src and Bcl-2 were upregulated in resected Eca109/NHE xenografts, whereas their expression was largely inhibited after ABT-737 and Dasatinib treatments in both Eca109/NHE and Eca109/Con grafts.

HE staining revealed a malignant phenotype, and IHC staining confirmed the expression of NHE9, phosphorylated Src, and Bcl-2. Histologic analysis revealed that tumor xenografts displayed the ESCC phenotype, and IHC staining showed increased expression of NHE9 in Eca109/NHE cells. Src phosphorylation and Bcl-2 expression were largely inhibited after treatment with Dasatinib and ABT-737 (Figure 7).

We concluded that NHE9-induced CRT resistance required Src/Akt/β-catenin activation and Bcl-2 upregulation. Additionally, the resistance could be weakened by the application of Src and Bcl-2 inhibitors.

### NHE9 expression and CRT response in ESCC patients

To determine the clinical relevance of NHE9 expression, we extended our analysis to an additional 105 ESCC patients who received the same regimen of neoadjuvant concurrent CRT described above. At the time of evaluation, a pathological complete response (pCR) and a non-pathological complete response (non-pCR) were achieved in 45 and 60 patients, respectively, with a therapeutic response rate of 42.9%. After CRT, all cases underwent esophagectomy performed by surgery teams supervised by experienced esophageal surgeons at Sun Yat-sen University Cancer Center.

**Figure 8:**
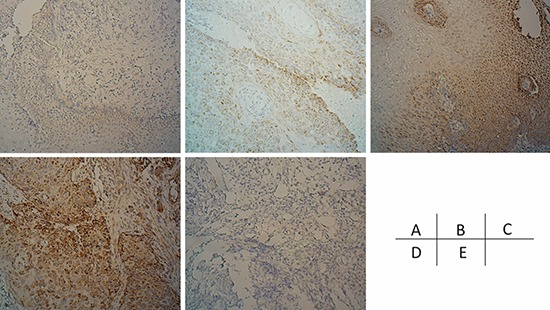
the expression of NHE9 in ESCC tissues ESCC cases demonstrating negative, weak, moderate, and strong NHE9 IHC signals are shown in addition to a negative control **A–E.**

An immunohistochemical assay was utilized to detect the expression of NHE9 in endoscopic biopsy samples before CRT. Results were obtained from 76 samples (samples could not be obtained from 26 patients, and inconclusive results were obtained from 3 slides). A total of 48 of the 76 patients showed strong positive staining (a score of 6–7), 22 patients showed moderate positive staining (a score of 3–5), and 6 patients showed weak positive staining (a score of 0–2). Positive immunoreactivity for NHE9 protein was mainly detected in the tumor cytoplasm; however, positive staining was also occasionally observed in the basal layer of the normal esophageal epithelium (Figure 8A–8D). Interestingly, strong positive staining was observed around microvessels, which might suggest a link between NHE9 and the tumor microenvironment.

We performed ROC curve analysis to develop a reasonable cut-off value for high NHE9 expression (Figure S3). Patients with a staining score of no more than 5 were placed in the “low expression” group, and the remaining patients were placed in the “high expression” group.

According to the cut-off value described above, high NHE9 expression was observed in 48 of 76 (63.16%) patients. The results of a Chi-square test revealed that higher NHE9 levels were associated with a poor pathological complete response (*P* < 0.001) and less changes in the T (*P* = 0.045) or N (*P* = 0.039) stage after CRT; however, no significant correlations were observed between the NHE9 expression level and other patient characteristics, including age, gender, BMI, tumor location, clinical stage, and lymph node stage change (Table 2).

**Table 2:** Association of NHE9 expression with ESCC patients’ clinicopathological features

Variable	Cases	NHE9 expression		*P* value#
		Low (%)	High (%)	
**Age**^1^				
** ≤55**	43	20 (46.5)	23 (53.5)	0.927
** >55**	33	15 (45.4)	18 (54.6)	
**Gender**				
** Male**	67	31 (46.2)	36 (53.8)	0.918
** Female**	9	4 (44.4)	5 (55.6)	
**Smoking**				
** Yes**	61	30 (49.2)	31 (50.8)	0.699
** No**	15	7 (46.7)	9 (53.3)	
**Alcohol**				
** Yes**	49	21 (42.9)	28 (57.1)	0.451
** No**	27	14 (51.9)	13 (48.1)	
**BMI**^2^				
** ≥22.5**	36	19 (52.8)	17 (47.2)	0.264
** <22.5**	40	16 (40.0)	24 (60.0)	
**Tumor location**				
** Upper**	10	5 (50.0)	5 (50.0)	0.943
** Middle**	50	22 (44.0)	28 (56.0)	
** Lower**	16	8 (50.0)	8 (50.0)	
**Clinical stage**				
** II**	16	6 (37.5)	10 (62.5)	0.440
** III**	60	29 (48.3)	31 (51.7)	
**pCR**				
** Yes**	33	5 (5.9)	28 (29.4)	<0.001*
** No**	43	30 (44.1)	13 (20.6)	
**T stage change**				
** 0–1**	31	10 (32.3)	21 (67.7)	0.045*
** 2–4**	45	25 (55.6)	20 (44.4)	
**N stage change**				
** −1–0**	29	9 (25.7)	20 (36.6)	0.039*
** 1–2**	47	26 (74.3)	21 (63.4)	

1 The mean age is 55

2 The mean BMI is 22.5

# Chi-square test

* statistical significance

## DISCUSSION

In this study, we uncovered an oncogenic role for NHE9. NHE9 overexpression enhanced the resistance of ESCC cells to apoptosis induced by cisplatin, vinorelbine or X-ray treatment. Conversely, NHE9 knockdown resulted in increased sensitivity to these therapies. In addition, the oncogenic function of NHE9 was further explored in a nude mouse xenograft model. We found that the overexpression of NHE9 induces CRT resistance in ESCC by upregulating the Src/Akt/β-catenin pathway and Bcl-2 expression. Inhibition of Src and Bcl-2 largely abolished the CRT resistance induced by NHE9. Taken together, these findings demonstrate that NHE9 may be an effective predictor of the response to CRT. Knowledge about NHE9 provides promising clues for enhancing CRT sensitivity in ESCC. These findings have significant clinical relevance.

Consistent with the experimental data *in vitro* and *in vivo*, higher expression of NHE9 was correlated with a poor CRT response and less decrease in T and N stage in ESCC patients, which further confirmed that NHE9 is a predictor of CRT resistance and ultimately poor survival. Therefore, knowledge about the expression level of NHE9 may help medical oncologists to select the best candidates for CRT.

RACK1 was identified as a binding partner of NHE9 in ESCC cells. Although the mechanism underlying the involvement of NHE9 in tumors was unclear, previous studies established an important role for RACK1 in cancer cell apoptosis [25, 27, 39, 40], which led us to investigate the possibility that NHE9 induced CRT resistance by affecting the RACK1-associated apoptosis pathway. Because RACK1 could affect cell apoptosis by regulating Src/Akt activity, we examined Src and Akt kinase activity under conditions of high or low NHE9 expression. Interestingly, we found that the interaction between NHE9 and RACK1 might be involved in CRT resistance. The binding affinity of NHE9 for RACK1 was much weaker after chemotherapy or radiation. Additionally, overexpression of NHE9 induced the upregulation of the Src/Akt/β-catenin pathway and Bcl-2 expression. Therefore, we presumed that an increase in the level of NHE9 protein might contribute to resistance by restoring the interaction between NHE9 and RACK1 and finally upregulating Src/Akt/β-catenin and Bcl-2.

Apoptosis is a pivotal mechanism in CRT-induced cell death. The balance among members of the Bcl-2 family of proteins determines the susceptibility of cells to a death signal in the intrinsic pathway [41]. Consistent with previous studies, the expression of Bcl-2, an anti-apoptotic member of the Bcl-2 family, was upregulated in cells exhibiting NHE9-overexpression-induced resistance to CRT. Src phosphorylation at Tyr416 was also identified in ESCC cells overexpressing NHE9. Phosphorylation of Tyr416 in the kinase domain upregulates enzyme activity and subsequently triggers a series of downstream kinase activation events, such as activation of Akt. The abnormal activation of Akt is an important mechanism underlying chemotherapy resistance in breast cancer [42], ovarian cancer [43], ESCC [44, 45], and lung cancer [46, 47]. Akt is activated by phosphorylation at Ser473 within its carboxy terminus, and it plays a role in cell survival by activating GSK-3β and downstream β-catenin. Upregulation of Bcl-2 expression and Src phosphorylation was also clearly observed in IHC images of Eca109/NHE9 nude mouse xenografts. To the best of our knowledge, our study is the first to reveal an oncogenic role for NHE9 and to elucidate the mechanism of CRT resistance in ESCC patients with increased NHE9 expression. Thus, Src/Akt/β-catenin and Bcl-2 might be effective therapeutic targets in ESCC patients with NHE9 expression-induced CRT resistance.

In the present study, we attempted to weaken the CRT resistance triggered by NHE9 expression by applying two targeted agents. Dasatinib, a Src kinase inhibitor, has been shown to be effective in treating solid tumors, such as thyroid cancer [48]. The Bcl-2 family inhibitor ABT-737 can effectively reduce chemotherapy resistance in breast cancer [49, 50]. Thus, we performed experiments to assess the therapeutic effects of these two targeted drugs on CRT resistance in ESCC. The results showed that Src phosphorylation and Bcl-2 expression were largely inhibited by the targeted inhibitors. Combining CRT with targeted-therapy with a single inhibitor led to an increased response to CRT in ESCC with higher NHE9 expression. Furthermore, the CRT response of grafts overexpressing NHE9 was restored when both targeted inhibitors were applied together with CRT. Currently, the application of targeted medication in ESCC is still in its infancy, and combined treatments including targeted drugs and CRT may be the best approach for extending patient survival. We hope to provide evidence for further clinical phase I trials of targeted therapies for ESCC.

In summary, this study provides not only new insights into the mechanisms of CRT resistance in ESCC but also clues regarding the application of new targeted agents in CRT-resistant ESCC patients. It is possible that NHE9 may weaken the response to CRT through other mechanisms aside from those described in this study; thus, further investigations should be carried out to better clarify the mechanisms underlying NHE9-induced CRT resistance with the aim of achieving a higher pathological response rate and better patient prognosis.

## MATERIALS AND METHODS

### Patients and tissue specimens

In accordance with the patient selecting criteria of microarray assay, another set of 105 patients diagnosed with stage II – III ESCC and undertook neoadjuvant concurrent CRT were consecutively selected from the department of Thoracic surgery, the Sun Yat-sen University Cancer Center. All the cases were selected based on availability of biopsy specimens and follow-up data. Patients included were without previous treatment, adjuvant chemotherapy or radiotherapy, malignant disease or a second primary tumor. All of the tumor samples from patients in this study were endoscopic biopsy specimens obtained before CRT. The preoperative workup was evaluated by endoscopy with biopsy and histological examination, barium esophagography to confirm tumor location, thoracic and abdominal computed tomography (CT) and cervical ultrasonography (with biopsy if indicated) if cervical lymph node metastasis was suspected. Endoscopic ultrasonography was performed routinely. All patients signed written informed consent before operation. The study was approved by the medical ethics committee of Sun Yat-sen University research institutes, Guangzhou, China.

All of the patients received the same concurrent CRT with the cisplatin / vinorelbine regimen. Cisplatin was administered as an i.v. drip at dose of 75 mg/m^2^ on day 1 and 22; vinorelbine 25 g/m^2^ was administered as a continuous i.v. drip for 48 hr on days 1, 8, 22 and 29. All patients received external beam radiotherapy by 6–8 MV linear accelerators. Two-dimensional or three-dimensional treatment plans using computed tomography scans were made. The initial treatment included the primary tumor and enlarged lymph nodes. For primary tumor, a radial margin of 1.5 cm and a proximal and distant margin of 3–4 cm were applied. A total radiation dose of 40 Gy (2 Gy/fraction, 5 days a week) was delivered with the three-field technique.

The effect of CRT was evaluated clinically for primary lesions based on esophagography and CT scan 4 weeks after CRT according to the following criteria: pCR was defined as the complete resolution of all assessable lesions. Otherwise, the patients were classified as non-pCR group. All these conditions had to last for at least 4 weeks and no appearance of new lesions.

Six to eight weeks after chemoradiotherapy, all patients received the right-approach tri-incisinal video-assistant thoracoscopic R0 esophagectomy with two-field systemic lymphadenectomy and cervical lymph node sampling. The alimentary tract was reconstructed using the gastric pull-up technique. The sites of the lymph nodes were identified by the surgeons during the operation.

The patients were followed every month for the first 3 month, every 3 months for the first year and then every 6 months for the next 2 years and finally annually. The diagnostic examinations consisted of esophagography, CT, chest X-ray, blood tumor biomarker test, and bone scan when necessary to detect recurrence and/or metastasis.

### Immunohistochemistry

IHC analysis was performed to examine NHE9 expression levels in endoscopic biopsy ESCC samples and nude mice graft tumor. Primary antibodies against NHE9 (1:200 dilution, anti-Rabbit, Abcam), phosphor-Src (Tyr416, 1:50 dilution, anti-rabbit, Cell signaling), and Bcl-2 (1:200 dilution, anti-rabbit, Cell signaling) were used in this study. NHE9 does not have any different isoform. Normal rabbit IgG-B (1:50 dilution, Santa) was used as negative control. HRP anti-rabbit/mouse antibody was used as secondary antibody (Dako). The intensity of IHC staining in the tumor cells was scored independently by two pathologists by using semiquantitative IRS (immunoreactive score) scale according to Remmele and Stegner [51]. The intensity of positive staining was scored according to the mean optical density method [52]:0, no staining; 1, weak staining (light yellow); 2, moderate staining (yellow brown); and 3, strong staining (brown). Positive tumor cell staining was assigned a score using a semiquantitative five-category grading system: 0, < 5% positive cells; 1, 5–25%; 2, 26–50%; 3, 51–75%; 4, 76–100%. The two individual parameters were added, resulting in an IRS ranging from 0 to 7. The value was selected until at least two pathologists reported consistent results.

Receiver operating characteristic (ROC) curve analysis was performed to determine cutoff score for NHE9 “high expression”. Tumors classified as low expression of NHE9 were those with the scores below or equal to the cutoff value, while tumors of high expression were those with scores above the value.

### RNA extraction, reverse transcription and real-time PCR

Total RNA was isolated from ESCC cell lines using TRIZOL reagent (Invitrogen, Carlsbad, CA). The extracted RNA was dissolved with RNAase-free water, and 1ug RNA from each sample was used for cDNA synthesis primed with random hexamers. Real-time PCR was performed using a Roche 480 fast real-time system (Applied Biosystems, Foster City, CA) to determine the expression pattern of NHE9 mRNA in each of the cell lines. Expression data were normalized to the geometric mean of the housekeeping gene glyceraldehydes 3—phosphate dehydrogenase (GAPDH). The first strand cDNA products were amplified with GAPDH-specific (F: 5′-CCACCCATGGCAAATTCCATGGCA-3′ and R: 5‘-TC TAGACGGCAGGTCAGGTCCACC-3′) and NHE9-specific (F: 5′-TTGCAATGGGGTCTGCGTAT-3′ and R: 5′-TTCCAGCATCGGGAACTCAC-3′) primers by PCR. The knockdown or overexpression efficiency was also evaluated.

### Plasmids

A complete protein coding region (CDS) of human NHE9 cDNA was cloned into pcDNA3.1 (Invitrogen) using EcoRI and Xho I sites. Subcloning was conducted into pMSCVpuro (Clontech) for stable transfection. A flag tag was added after NHE9 for immunoprecipitation assay. C-terminal of NHE9 was clone into pGEX-6p-1 (GE Healthcare), for protein prokaryotic expression and following pull-down assay. pEGFP-C2-NHE9 was constructed for fluorescein observation, using pEGFP vector (Clontech). The shRNA sequences targeted NHE9 are listed as following:, (1) GCTCTTCAGAATGTGGTAT (loop) ATACCACATTCTGAAGAGCCG; (2) ATCGTCATAGGGTTAATTA (loop) TAATTAACCCTATGACGATGC. pSUPER.retro vector (Oligoengine) was used for construction of short interfering RNA. Packaging plasmid pik (Langri biotechnology, Guangzhou) was used in virus construction. The complete CDS regime of human RACK1, GSTP1, LIGO4, PKD1 cDNA was cloned into pcDNA6/myc-HisB (Invitrogen) for co-immunoprecipitation assay.

### Overexpression or knock down of NHE9

pMSCV-NHE9 and pMSCV vector were transfected into Eca109 and KYSE30 to construct stable NHE9 overexpression and control cell line; pSUPER.retro-shNHE9 and pSUPER.retro vector were used to produce retrovirus and then constructed stable NHE9 knocking down and control cell lines. Packaging of viruses was performed by transient transfection of 293FT cells with a transfer plasmid and pik packaging vector. Seventy-two hours after transfection, the retrovirus particles were collected and filtered, then concentrated by ultracentrifugation at 50, 000 g for 2.5 hr at 4ºC. Subsequently, we infected the esophageal cancer cell lines Eca109, KYSE30, KYSE180 and KYSE520 with the retrovirus in 6-well plates. 48 hours after infection, DMEM medium with 1.5ug/ml puromycin was applied to select the successful transfected cells. The overexpression or knockdown efficiency of NHE9 was examined by real-time qPCR and Western blotting.

### Transient transfection

RACK1, CHP1, CHP2, GSTp1, LIGO4, and PKD1 cDNA was cloned into pcDNA6/myc-HisB vector and transfected to produce overexpression cell lines. The KYSE30/NHE9 and Eca109/NHE9 cells were prepared for RACK1 transfection.

### Western blotting analysis

Protein samples were resolved by SDS-polyacrylamide gel electrophoresis and electrotransferred on a polyvinylidene difluoride membrane (Pall Corp., Port Washington, NY). The samples were then incubated with primary antibodies against NHE9 (1:800 dilution, Abcam, Cambridge, MA), GAPDH (1:5000 dilution, KangChen, Shanghai), β-actin (1:1000 dilution, Cell signaling), Caspase 3 (1:1000 dilution, Cell signaling), phosphor-Akt (1:1000 dilution, Cell signaling), phosphor-Src (Tyr416, 1:1000 dilution, Cell signaling), PARP (1:1000 dilution, Cell signaling), β-catenin (1:1000 dilution, BD), pGSK3β (1:1000 dilution, Cell signaling), Bcl-2 (1:1000 dilution, Cell signaling), Flag-tag (1:1000 dilution, Sigma), c-Myc (1:200 dilution, Santa), normal mouse IgG (1:200 dilution, Santa). The secondary antibodies used were ECL^TM^ anti-mouse IgG (1:2500 dilution, GE healthcare) and ECL^TM^ anti-rabbit IgG (1:2500 dilution, GE healthcare). The immunoreactive signals were detected with enhanced chemiluminescence kit (Amersham Biosciences, Uppsala, Sweden). The procedures followed were conducted in accordance with the manufacturer's instructions.

### X-ray survival assay

Cells were dispensed in 6-wells cell culture dishes at a density of 1 × 10^3^ cells. After 24 h incubation, they were irradiated with x-ray (6 J/m^2^, x-ray irradiation was carried out using x-ray machine for research only), and the cultures were maintained until the surviving cells formed colonies. The colonies that survived after incubation were then stained with crystal violet, counted, and relative colony numbers were obtained.

2 × 10^5^ cells (cover 50% area of the dish) were cultured in 60mm dish for 24 hours, and then irradiated with x-ray (6 J/m^2^). The cells were collected after 48 hours for study of protein and mRNA expression alteration.

### Chemotherapy resistance assay

To assess chemosensitivity to anti-cancer drugs, cells were dispensed in 96-well plates at a density about 3 × 103 cells per well and incubated with a range of different concentrations of cisplatin or vinorelbine for 72 hours. Cell viability values were assessed by the MTT (3- (4, 5-Dimethylthiazol-2-yl)-2, 5-diphenyltetrazolium bromide, inner salt) method (Cell Titer 96 Aqueous One Solution Cell Proliferation Assay solution; Promega, Madison, WI). Spectrometric absorbance at wavelength of 570 nm and 655 nm were measured on a microplate reader (Bio-Rad). Cell viability was evaluated with the value of [A570-A655 (drug+)/A570-A655 (drug-)] × 100%.

2 × 10^5^ cells (cover 50% area of the dish) were cultured in 6-well cell culture dishes for 24 hours, and then they were treated with cisplatin (1 ug/ml) or vinorelbine (1.5 × 10^−2^ug/ml) for 48 hours. The cells were collected for studying protein and mRNA expression changes.

### Flow cytometry

To quantify the apoptotic cells, the occurrence of apoptosis was determined by staining cells with both annexin V-fluorescein isothiocyanate and propidium iodide (PI). The apoptosis assay was conducted using the protocol according to manufacturer's instructions (BioVision, Milpitas, CA). Fluorescence-activated cell sorting (FACS) was performed with a Beckman Dickinson FACSort apparatus and used to quantitate the apoptotic population based on DNA levels.

### Pulldown assay

NHE9 c-terminal (NHE9^c^) was expressed as a complex of a glutathione S-transferase (GST)-fused human SLC9A9. Recombinant proteins fused with GST binding protein were expressed in BL21 Star (DE3) cells. The protein complex was induced with isopropyl-β-D-thiogalactopyranoside (IPTG) and purified with glutathione-Sepharose (Sigma). Bacterial from 500 ml of LB-medium (MP biomedicals, LLC) culture were sonicated and centrifuged, and the supernatant was applied to an amylose resin column equilibrated with phosphate-buffered saline (PBS) and incubated for 30 min at room temperature. The resin was washed 3 times with PBS, and cell lysate of Eca109 (10 dishes, 10 cm) was added to the resin. After incubation for 1 h at 4°C, the resin was washed 6 times with PBS, and bound proteins were eluted with sample buffer and analyzed by SDS-PAGE. The positive results were analyzed with surface- enhanced laserdesorption/ionization – time of flight – mass spectrometry (SELDI- TOF- MS) to detect the proteins. The same sample also prepared for western blotting identification using the corresponding antibodies.

### Immunoprecipitation

1 × 10^8^ NHE9 stable transfectants or mock controls were lysed in lysis buffer (20 mM Tris, pH 7.5, 150 mM, NaCl, 1% Triton X-100, 1 mM EDTA, 10ug/ml aprotinin, 10 ug/ml leupeptin, and 1 mM phenylmethylsulfonyl fluoride. Cell lysates were incubated with anti-Flag beads (Sigma) or anti-Myc antibodies (Santa Cruz Biotecnology) or control normal mouse IgG (Santa Cruz Biotecnology). The immune complex was precipitated using Protein-A/G-agarose beads (when using anti-Myc or normal mouse IgG in IP) and resolved by SDS-PAGE. The blot was then probed with anti-FLAG (Sigma) or anti-Myc antibodies (Santa Cruz Biotecnology), respectively.

### Nude mice xenograft models

The orthotropic nude mice esophageal squamous cell carcinoma model was established with human esophageal cancer cell Eca109/NHE and Eca109/Con. 10^6^ cells in 150 ul of culture medium were subcutaneously injected into the left (Eca109/NHE) or right flank (Eca109/Con) of five-week old female *nu/nu* mice maintaining in standard SPF conditions. When tumor volume reached approximately 300 mm^3^, the long and short diameter of each graft tumor were recorded and tumor volume was quantified with the value of L*W^2^/2 (L, long diameter; W, short diameter).

The first time cisplatin + vinorelbine intraperitoneal injection started when tumor volume reach 300 mm^3^. The dosage for mice was calculated using HD/HBSA*MBSA (HD, human dosage; HBSA, human body surface area; MBSA, mouse body surface area). So cisplatin+ vinorelbine were injected every five days, 4 times, with a dose of 0.04 mg/20g (5.78mg/m^2^) and 0.02 mg/20 g (2.9 mg/m^2^); meanwhile 0.9% normal saline was injected as negative control. Dasatinib (Selleck, S1021 or ABT-737 (Selleck, S1002) was injected 0.4 mg/20 g in 0.1ml PBS intraperitoneal in combination with cisplatin and vinorelbine. Combined therapy of Dasatinib+ ABT-737 was performed by injecting a mix of 0.4 mg/20 g of each inhibitor. No less than 5 mice were performed for each group.

The x-ray radiation (6 J/m^2^) was performed when tumor volume reach 500 mm^3^. Mice holder was used during radiation. The tumor was well exposed, while other part of mice body was carefully protected using plumbum mould. No less than 5 mice were performed for each group. The study had been licensed by experimental animal care commission of Sun Yat-sen University, according to the regulations of experimental animal management of Guangdong Province.

### Statistical analysis

Statistical analysis was performed with SPSS software (SPSS Standard version 19.0, SPSS, Chicago, IL). The Chi-square test or Fisher's exact test was applied to evaluate the correlation of NHE9 and clinicopathological parameters. The ROC curve was performed using MedCalc for Windows, version 11.4 (MedCalc Software, Ostend, Belgium). The experimental data represent at least three independent tests. Statistical comparisons were made using Students’*t*-test and One-way ANOVA analysis. *P* < 0.05 were considered to represent a statistically significant difference.

## SUPPLEMENTARY FIGURES


