# Supplementary material for: NHE9 induces chemoradiotherapy resistance in esophageal squamous cell carcinoma by upregulating the Src/Akt/β-catenin pathway and Bcl-2 expression
Source: Oncotarget. 2015 Apr 10;6(14):12405–20. doi: 10.18632/oncotarget.3618 (PMC4494947; doi:10.18632/oncotarget.3618)
Supplement: Supplementary file 1 [file oncotarget-06-12405-s001.pdf]

SUPPLEMENTARY FIGURES

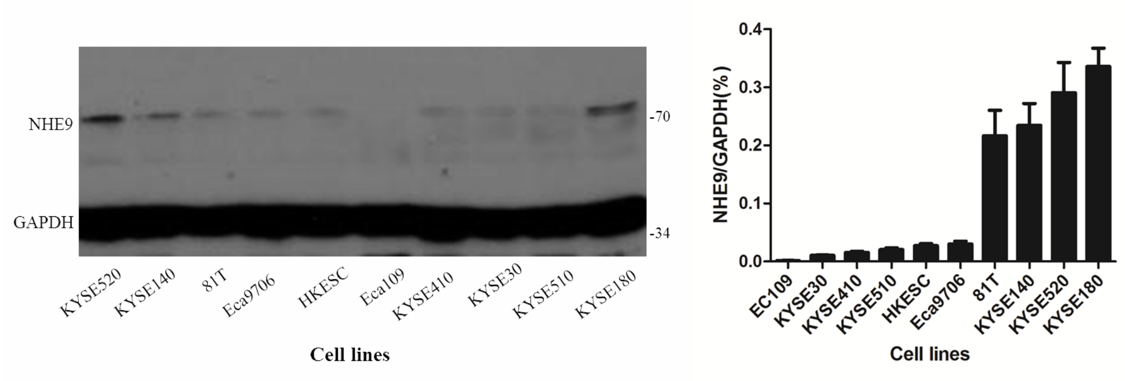

Supplementary Figure S1: Intrinsic NHE9 expression levels in ten ESCC cell lines. NHE9 expression levels were evaluated by western blot **A.** and real-time qPCR **B.**

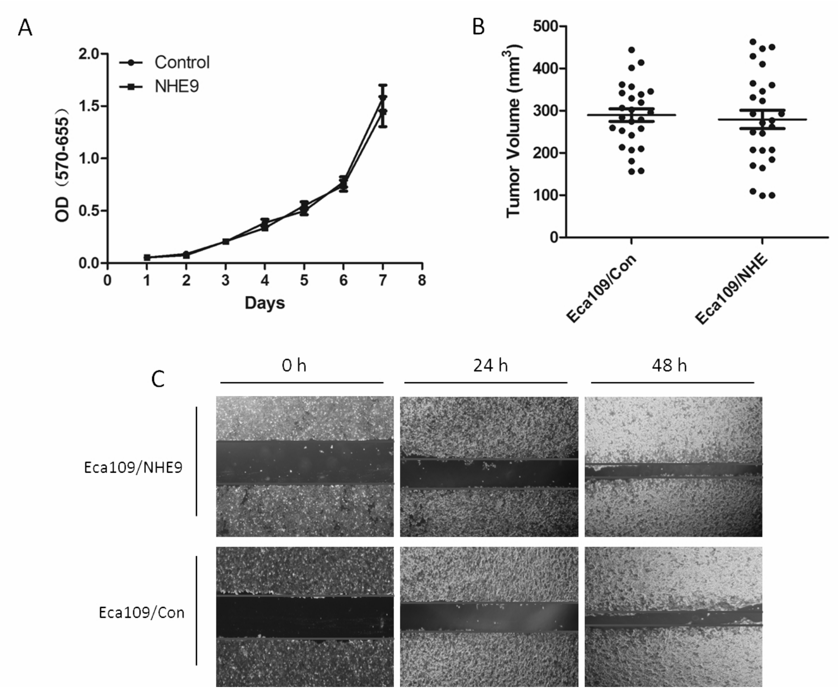

Supplementary Figure S1: NHE9 has no effects on the growth, proliferation or migration of ESCC cells. No apparent difference was observed in the cell growth curve **A.** and the tumor volume was similar between Eca109/NHE and Eca109/Con grafts **B.** Additionally, NHE9 did not exhibit effects on cell migration in a wound-healing assay **C.**

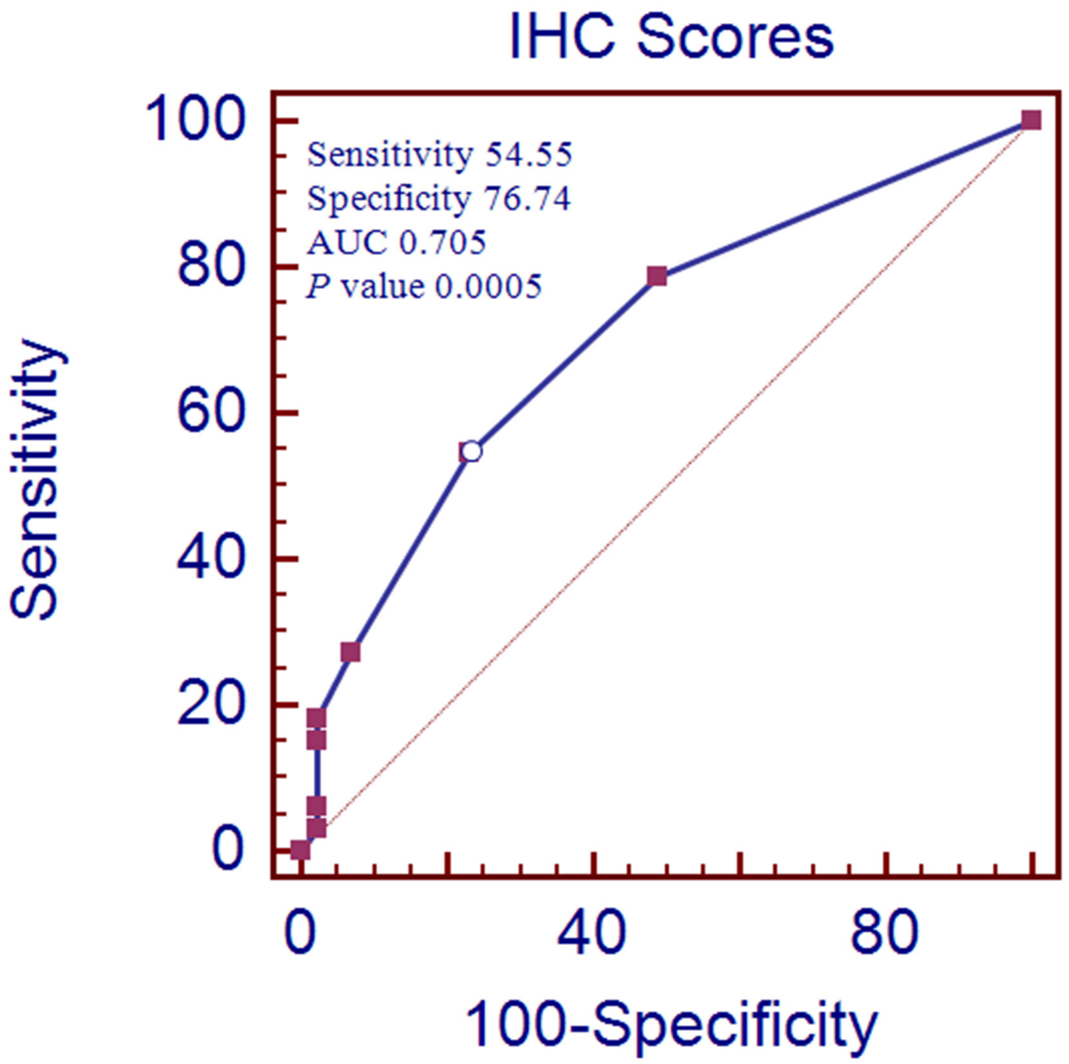

Supplementary Figure S3: ROC curve analysis of NHE9 IHC scores.
